# Green Synthesis of TiO_2_ Nanoparticles Using *Annona muricata* Extract and Their Application as CO_2_ Corrosion Inhibitor for 1018 Carbon Steel

**DOI:** 10.3390/ma19194201

**Published:** 2026-10-01

**Authors:** Alfredo Brito-Franco, Diana Francisco-Rojas, Ana Karen Galvez-Larios, Edna Vázquez-Veléz, America María Ramírez-Arteaga, Roy López-Sesenes, José Gonzalo González-Rodriguez

**Affiliations:** 1 Centro de Investigaciones en Ingeniería y Ciencias Aplicadas, Universidad Autónoma del Estado de Morelos, AV. Universidad 1001, 62209 Cuernavaca, Mor, Mexico; karengalvex@gmail.com (A.K.G.-L.); ggonzalez@uaem.mx (J.G.G.-R.); 2 Facultad de Ciencias Químicas e Ingeniería, Universidad Autónoma del Estado de Morelos, AV. Universidad 1001, 62209 Cuernavaca, Mor, Mexico; diana.franciscor@fcqei.uaem.edu.mx (D.F.-R.); america.rmz@uaem.mx (A.M.R.-A.); rlopez@uaem.mx (R.L.-S.); 3 Instituto de Ciencias Físicas, Universidad Nacional Autónoma de Mexico, AV. Universidad 1001, 62209 Cuernavaca, Mor, Mexico; velez.edna@icf.unam.mx

**Keywords:** carbon steel, CO_2_ corrosion, green synthesis, nanoparticles

## Abstract

The corrosion inhibition performance of green-synthesized TiO_2_ nanoparticles on CO_2_-induced corrosion of 1018 carbon steel was evaluated using potentiodynamic polarization, linear polarization resistance (LPR), and electrochemical impedance spectroscopy (EIS) techniques. The polarization results revealed that TiO_2_ nanoparticles behave as an effective mixed-type inhibitor, with inhibition efficiency increasing with concentration and reaching its maximum at 600 ppm. Thermodynamic analysis demonstrated that the adsorption process deviates from ideal behavior and accurately fits Kokalj’s general-purpose Type-1 isotherm, involving a spontaneous, combined physisorption–chemisorption mechanism. Their presence facilitates the formation of a protective film, as demonstrated by lower corrosion current densities and higher charge transfer resistance values as compared to the uninhibited system. Specifically, the corrosion current density decreased by one order of magnitude, while the polarization resistance showed a notable increase upon nanoparticle addition. Similarly, the impedance modulus increased as a result of nanoparticle adsorption. In the absence of the inhibitor, the steel exhibited high susceptibility to severe uniform corrosion. In contrast, surface degradation was significantly reduced when TiO_2_ nanoparticles were present. However, at higher concentrations (800 ppm), structural defects and localized pitting were observed, likely due to nanoparticle agglomeration.

## 1. Introduction

Carbon steel, particularly AISI 1018 grade, is extensively used as a primary construction material in the hydrocarbon extraction and transportation industry due to its favorable mechanical properties and cost-effectiveness. Consequently, the structural integrity and reliability of infrastructure in this sector are closely linked to the durability of these materials [1].

However, in oil and gas operations, the presence of acidic gases such as CO_2_ and H_2_S—commonly utilized in enhanced oil recovery—creates highly aggressive service environments [2,3,4]. Among the resulting degradation mechanisms, sweet corrosion is especially critical. This process originates from the hydration of CO_2_ to form carbonic acid (H_2_CO_3_), which accelerates anodic metal dissolution while promoting cathodic hydrogen evolution [5].

Although the formation of corrosion products, primarily iron carbonate (FeCO_3_), may provide a certain degree of passivation, the presence of chloride ions can destabilize this protective layer. This destabilization enhances corrosion processes, significantly reducing pipeline service life [6]. As a consequence, operational safety is compromised, leading to substantial economic losses, which are estimated to represent approximately 1% to 5% of the global Gross Domestic Product (GDP) due to mitigation, maintenance, and material replacement costs [7,8].

One effective strategy to mitigate metal degradation is the use of inorganic corrosion inhibitors, a practical and widely implemented approach for the internal protection of pipelines [9]. Nevertheless, increasingly stringent environmental regulations, such as those established by OSPAR, have driven a shift away from toxic synthetic inhibitors toward more environmentally sustainable alternatives [10]. In this context, significant research efforts have focused on the development of low-cost, eco-friendly inhibitors free of heavy metals, including those derived from plant extracts, fruit extracts, and biopolymers [11,12]. Despite their advantages, these green inhibitors often exhibit notable limitations, such as insufficient surface coverage, a limited number of active adsorption sites, low solubility, and poor stability under varying environmental conditions [13]. To overcome these challenges, nanotechnology-based strategies have been introduced to enhance their anticorrosion performance across a wide range of aggressive environments [14,15].

The use of metal oxide nanoparticles has attracted growing interest as an effective strategy for corrosion inhibition due to their thermodynamic stability, low toxicity, and high surface-to-volume ratio. To effectively mitigate corrosion, dispersed nanoparticles must successfully adsorb and remain retained on the metallic substrate, a process heavily governed by electrostatic interactions and colloidal stability [16]. In a CO_2_-saturated 3.5 wt.% NaCl solution, the working pH is mildly acidic (typically around 4). Since the point of zero charge (PZC) of titanium dioxide (TiO_2_) generally lies between pH 5.8 and 6.8, the nanoparticles acquire a net positive surface charge in this medium [17]. This positive charge promotes strong electrostatic attraction toward the steel surface, which is often negatively charged due to the specific pre-adsorption of chloride ions. However, the high ionic strength of the electrolyte compresses the electrical double layer of the dispersed particles, which can compromise colloidal stability and induce agglomeration at high concentrations [18]. Despite this agglomeration tendency in the bulk solution, once the nanoparticles are successfully retained at the metal/electrolyte interface, the inhibition process operates via two distinct mechanisms. First, active-site blocking alters electrode kinetics by adsorbing onto specific anodic and cathodic regions, directly reducing the charge transfer rates. Second, the subsequent accumulation of nanoparticles forms a structural diffusion barrier, which is a mass transport phenomenon that physically restricts the migration of aggressive species (e.g., Cl^−^, H_2_CO_3_ and H^+^) toward the bare metal [19,20].

Among the various metal oxides, TiO_2_ stands out due to its high chemical stability, low toxicity, and semiconducting properties [21,22]. While TiO_2_ has been extensively studied in photocatalytic applications and as a reinforcing agent in passive coatings, recent research highlights its potential as a corrosion inhibitor in aqueous environments. In addition, green synthesis routes employing plant extracts as reducing and stabilizing agents eliminate the need for toxic solvents, making the process more environmentally sustainable [23,24].

The synthesis of TiO_2_ nanoparticles (NPs) using a wide range of plant extracts—including *Mentha arvensis* leaves [25], *Trigonella foenum-graecum* extract [26], *Moringa oleifera* [27], and banana peel [28]—has been widely reported. These studies indicate that the polyphenolic compounds present in such extracts not only facilitate the reduction of titanium ions and stabilize the anatase crystalline phase, but also remain adsorbed on the nanoparticle surface. This residual adsorption enhances the interaction between the nanoparticles and the metallic substrate [29,30,31].

Shafaamri et al. [32] investigated the effect of TiO_2_ nanoparticles on the corrosion resistance of an epoxy-based coating system in a 3.5% NaCl solution using electrochemical impedance spectroscopy (EIS). Their results showed that the incorporation of TiO_2_ improved the protective properties of the coating and increased its hydrophobicity, with optimal performance observed at 0.1 wt.% TiO_2_.

In another study, Zhu et al. [33] evaluated the behavior of TiO_2_ nanoparticles in aggressive environments containing CO_2_, H_2_S, and mixed CO_2_/H_2_S systems. The results revealed that chloride ions could penetrate the electrochemical double layer formed between the metal surface and the nanoparticles, leading to the breakdown of the oxide film. This process accelerated corrosion, with the most severe degradation observed in H_2_S-containing environments.

Despite the growing interest in nanoparticle-based corrosion inhibitors, there is a critical need to understand their stability and fundamental interactions in aggressive media. Specifically, the synergistic effect of phytochemical capping agents from *Annona muricata* on the colloidal stability, inter-particle agglomeration, and subsequent adsorption kinetics of TiO_2_ nanoparticles in high ionic strength, acidic CO_2_ environments remains unknown. Therefore, the present study aims to evaluate the corrosion inhibition performance of TiO_2_ nanoparticles on AISI 1018 carbon steel in a 3.5 wt.% NaCl solution saturated with CO_2_ at 50 °C.

## 2. Materials and Methods

### 2.1. Test Material

Working electrodes were fabricated from commercial AISI 1018 carbon steel (Distribuidora de Aceros y Metales DAM, Cuernavaca, Mexico). The chemical composition of this alloy is (in wt.%): 0.18 C, 0.75 Mn, 0.04 P, 0.05 S, with Fe as the balance. Prior to all electrochemical assessments, the active surface area of the specimens was mechanically abraded utilizing silicon carbide papers up to 600-grit. Immediately after grinding, the electrodes were sequentially degreased with acetone (Meyer, Mexico City, Mexico), rinsed with double-distilled water, and dried under a warm air stream.

### 2.2. Test Solution

The corrosive environment consisted of a 3.5 wt.% NaCl solution prepared from analytical-grade reagents (Meyer, Mexico City, Mexico) and double-distilled water. The temperature of the electrolyte was maintained at 50 °C using a hot plate. To ensure full saturation and remove dissolved oxygen, the solution was continuously purged with CO_2_ gas (Infra, Cuernavaca, Mexico) for 2 h prior to immersing the working electrodes, maintaining the gas flow throughout the entirety of the experiments. Under these saturated conditions, the formation of carbonic acid acidified the electrolyte, and the pH of the testing solution was experimentally measured to stabilize at approximately 3.9.

### 2.3. Green Synthesis of TiO_2_ NPs

Fresh leaves of *Annona muricata* were collected from Xoxocotla, Morelos, Mexico in May 2026. The taxonomic identification of the plant material was authenticated by botanist Columba Monroy Ortiz, and a voucher specimen has been deposited in the Herbarium HUMO-CIBYC-UAEM under the voucher reference number 41846. Upon collection, the foliar biomass was subjected to rigorous washing, air-drying, and mechanical milling to yield a homogenous powder. An aqueous infusion was prepared by suspending 20 g of this biomass in 100 mL of deionized water, subjecting the mixture to controlled heating at 80 °C for 120 min. The mixture underwent vacuum filtration to isolate the bioactive aqueous phase. The nanoparticle synthesis was initiated by combining equal volumes (100 mL each) of the *Annona muricata* infusion and a 0.5 M titanium tetrachloride (TiCl_4_) precursor solution (Sigma-Aldrich, St. Louis, MO, USA). This reaction mixture was continuously agitated for 3 h under ambient conditions. A visible colorimetric transition to a light-brown hue served as the primary indicator for the successful biologically assisted hydrolysis of the titanium precursor and the subsequent nucleation of the nanoparticles. Unlike the synthesis of metallic nanoparticles where plant extracts act as reducing agents, the formation of TiO_2_ from TiCl_4_ proceeds via a hydrolysis and polycondensation mechanism, as the titanium ion remains in the Ti^4+^ oxidation state. The overall chemical reaction can be expressed as follows:

(1) $$TiCl_{4}+2H_{2}O\overset{A.\;\mathrm{muricata}\;\mathrm{extract}}{\to }TiO_{2}+4HCl$$

In this process, the complex phytochemicals present in the *Annona muricata* extract act as potent capping and stabilizing agents, controlling the growth of the TiO_2_ nuclei and preventing their complete agglomeration [34].

The synthesized nanomaterial was recovered via centrifugation at 4000 rpm for 15 min. To ensure the elimination of unreacted precursors and byproducts, the pellet was sequentially washed with deionized water and absolute ethanol. The final recovery involved a desiccation step at 80 °C for 24 h, followed by a thermal calcination process at 400 °C for 3 h to promote the crystallization of the TiO_2_ nanoparticles. Physicochemical and morphological characterization of the calcined material was conducted through a multi-analytical approach. Crystallographic profiling was executed utilizing a Bruker D2 Phaser X-ray diffractometer (Bruker AXS, Karlsruhe, Germany), employing Cu Kα radiation (λ = 1.54 Å) over a 10 min acquisition window. Elemental composition was verified via X-ray fluorescence (XRF) spectroscopy utilizing a Bruker XRF spectrometer (Bruker AXS, Karlsruhe, Germany). Nanoscale morphological features and particle dimensions were resolved using a Zeiss Transmission Electron Microscope (TEM) (Carl Zeiss, Oberkochen, Germany) configured at an acceleration voltage of 100 kV. Prior to TEM imaging, a dilute suspension was prepared by sonicating 2.5 mg of the nanopowder in 5 mL of absolute ethanol (Meyer, Mexico City, Mexico), from which a 5 μL droplet was cast onto a standard carbon-coated copper grid.

### 2.4. Electrochemical Techniques

All corrosion evaluations were conducted in a conventional glass cell equipped with a three-electrode array. The epoxy-mounted AISI 1018 steel, with an exposed active surface area of 0.3166 cm^2^, functioned as the working electrode, while a graphite rod and an Ag/AgCl electrode were employed as the auxiliary and reference electrodes, respectively.

To ensure the reliability of the electrochemical data and avoid cross-contamination or history effects, independent test sequences were performed on fresh AISI 1018 steel specimens. All electrochemical measurements were conducted at least in triplicate under identical experimental conditions to guarantee statistical reproducibility.

For long-term continuous monitoring, Open Circuit Potential (OCP), Linear Polarization Resistance (LPR), and Electrochemical Impedance Spectroscopy (EIS) were evaluated sequentially on the same set of specimens over a 24 h immersion period. LPR measurements were performed by polarizing the working electrode within ±15 mV around the *E_corr_* at 60 min intervals. Concurrently, EIS spectra measurements were performed at *E_corr_* using an AC perturbation amplitude of ±10 mV (peak-to-peak) across a frequency range from 10 kHz to 0.01 Hz.

For the potentiodynamic polarization evaluations, a completely separate set of fresh specimens was utilized to prevent prior surface alteration. After an initial OCP stabilization period of 2 h, the anodic and cathodic branches were recorded independently on fresh specimens. This approach was adopted because a single continuous scan starting from strong cathodic overpotentials would artificially alter the surface chemistry and degrade the biogenic inhibitor film prior to anodic dissolution. Consequently, the separate scans were performed starting from the stabilized *E_corr_* up to +250 mV (anodic branch) and from *E_corr_* down to −250 mV (cathodic branch) at a scan rate of 1 mV/s. The corrosion current density ($i_{corr}$) was obtained by Tafel extrapolation, and the inhibition efficiency (I.E.) was calculated using the following equation:

(2) $$I.E.\left(\%\right)=\frac{i_{corr1}-i_{corr2}}{i_{corr1}}\times 100,$$

where *i_corr1_* and *i_corr2_* denote the corrosion current densities measured in the absence and presence of TiO_2_ nanoparticles, respectively.

All electrochemical measurements were recorded using an ACM Instruments potentiostat(ACM Instruments, Grange-over-Sands, UK). Electrochemical test conditions including LPR and EIS, were conducted in accordance with ASTM G59 [35] and ASTM G106 [36] standards, respectively, as well as previously reported methodologies for sweet corrosion environments [37].

## 3. Results and Discussion

### 3.1. TiO_2_ Nanoparticles Characterization

#### 3.1.1. XRD

**Figure 1:**
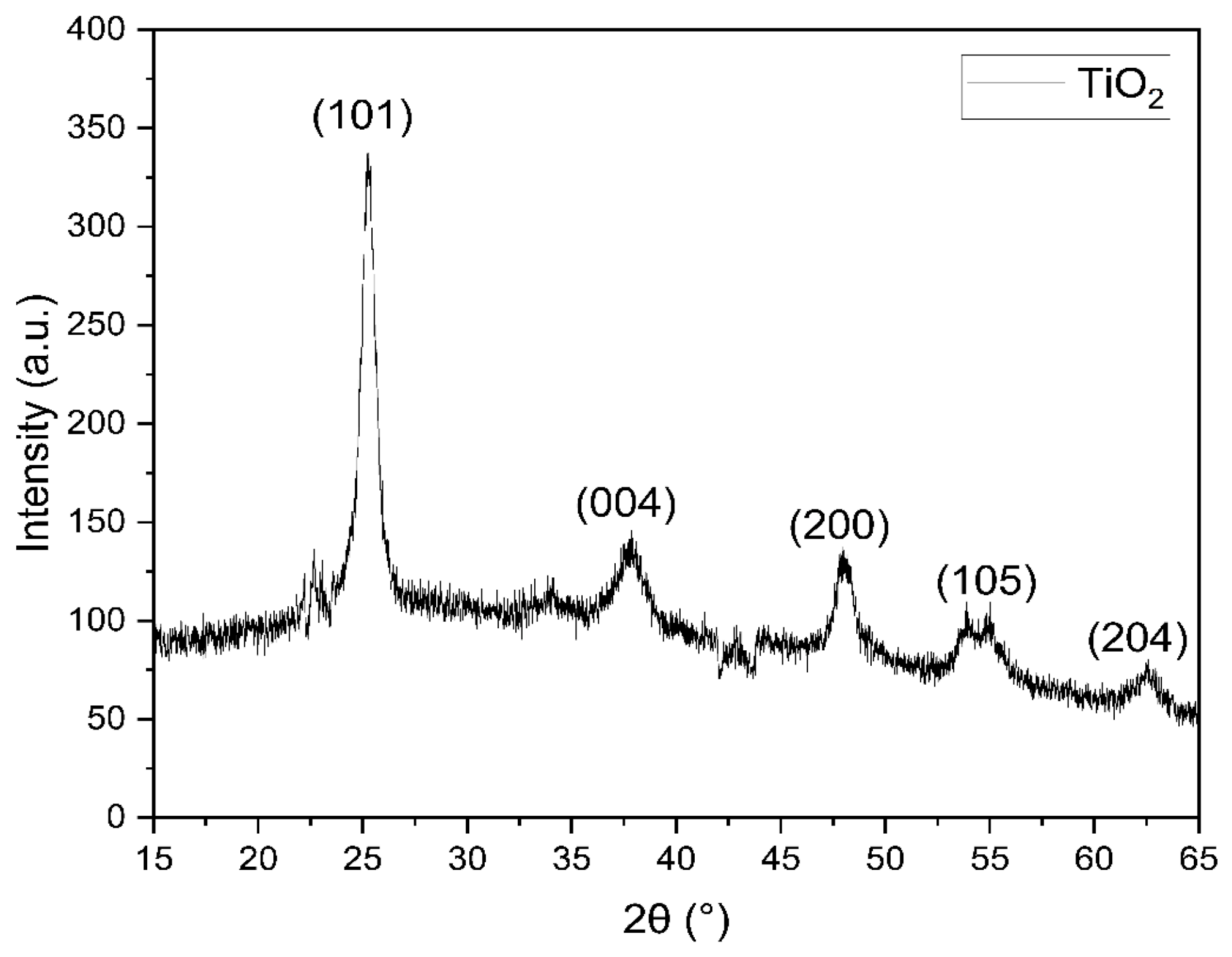
X-ray diffraction patterns of the green-synthesized TiO_2_ NPs, confirming the anatase crystalline phase (JCPDS No. 21-1272).

The X-ray diffraction (XRD) pattern of TiO_2_ nanoparticles synthesized using an aqueous extract of *Annona muricata* leaves is illustrated in Figure 1. The diffractogram shows distinct peaks at 2θ values of 25.28°, 37.80°, 48.05°, 53.88°, and 62.68°, which are assigned to the (101), (004), (200), (105), and (204) crystallographic planes, respectively. These peaks confirm the formation of TiO_2_ nanoparticles with a tetragonal crystal structure corresponding to the anatase phase (JCPDS file No. 21-1272). Furthermore, no additional peaks associated with other crystalline phases, such as rutile or brookite, or with impurities were observed, demonstrating the high phase purity of the synthesized TiO_2_ nanoparticles [38,39].

#### 3.1.2. TEM

**Figure 2:**
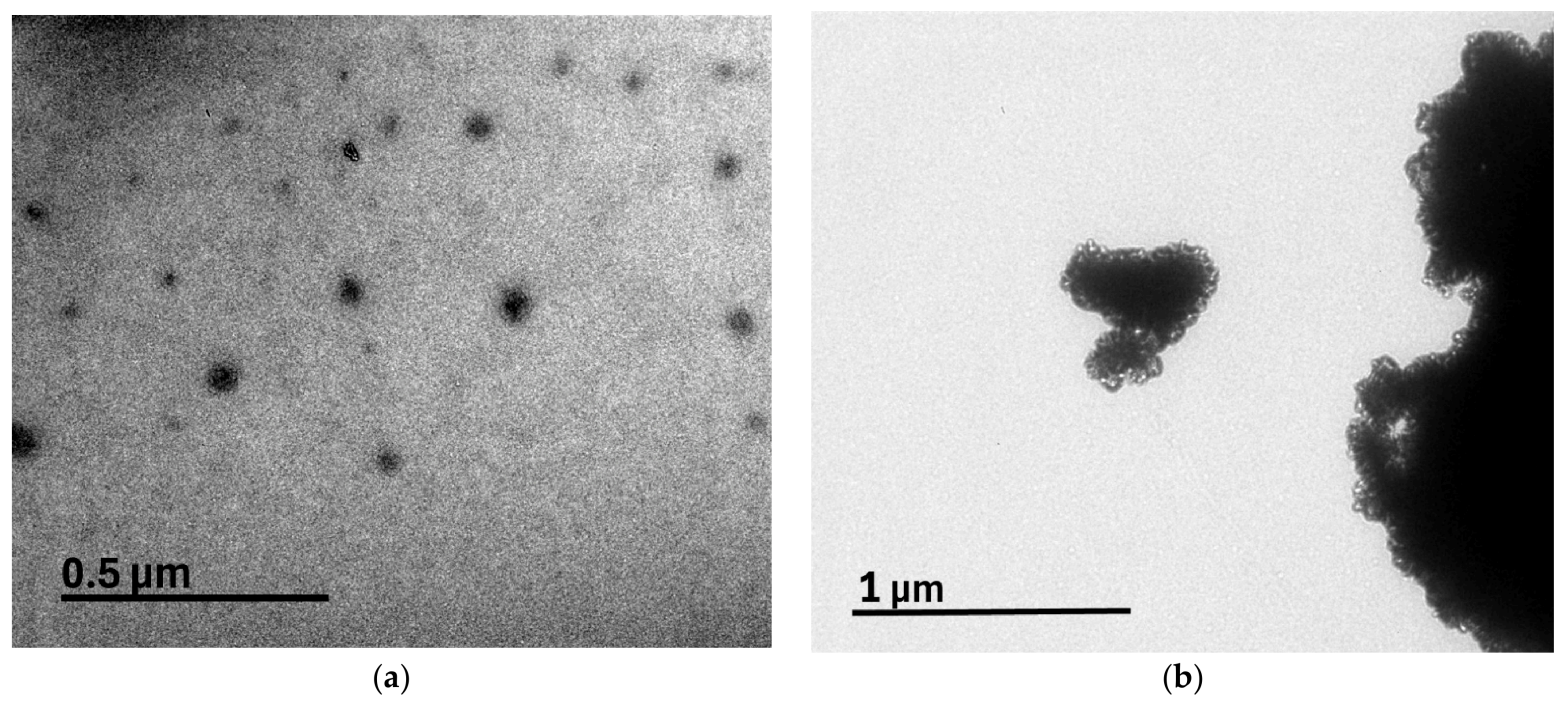
TEM micrographs of the green-synthesized TiO_2_ NPs: (**a**) spherical morphology and individual particle size, and (**b**) formation of irregular agglomerates.

The morphology and particle size of the TiO_2_ nanoparticles were characterized by TEM, as presented in Figure 2. The image in Figure 2a shows that the nanoparticles are predominantly spherical. To determine the particle size accurately, image processing software (ImageJ version 1.54g) was used to measure individual nanoparticles, yielding an average diameter of 31.9 ± 8.7 nm. Moreover, Figure 2b highlights the tendency of the TiO_2_ nanoparticles to form irregular agglomerates. Rather than an absence of stabilizing agents, this aggregation behavior is primarily attributed to the inherently high surface energy of nanoparticles and the thermal calcination process at 400 °C. Although the phytochemicals from the *Annona muricata* extract act as capping agents that restrict initial overgrowth during the liquid phase, the subsequent drying and high-temperature calcination steps promote the partial sintering and clustering of the nanoparticles to reduce their overall thermodynamic surface energy [40,41,42].

#### 3.1.3. XRF

**Figure 3:**
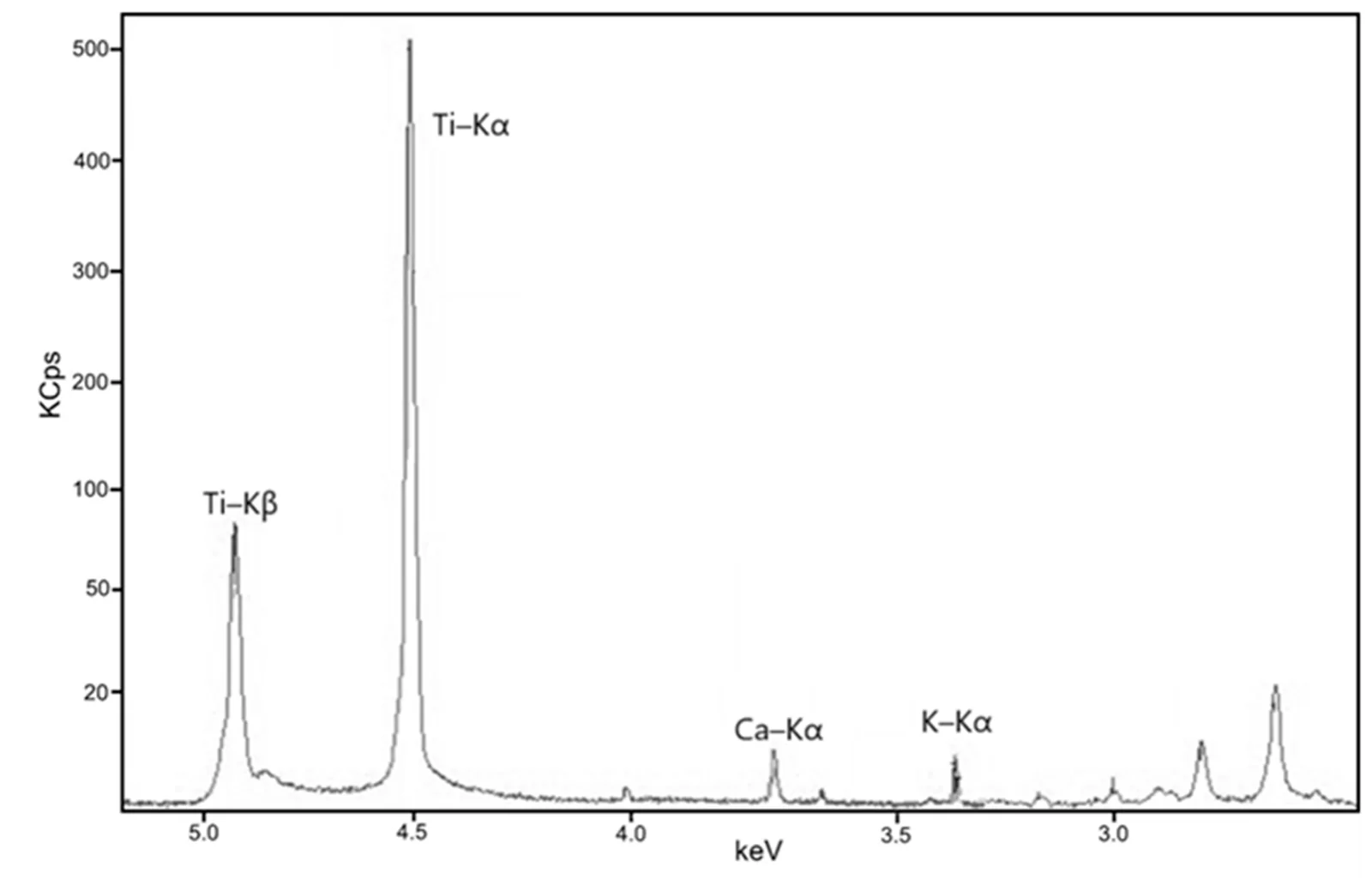
XRF spectrum of the green-synthesized TiO_2_ NPs.

The elemental composition and purity of the green-synthesized TiO_2_ nanoparticles were analyzed by X-ray fluorescence (XRF) spectroscopy. The corresponding spectrum for the TiO_2_ nanoparticles obtained using *Annona muricata* extract is presented in Figure 3. The spectrum exhibits intense characteristic peaks associated with titanium. A prominent emission peak appears at 4.51 keV, corresponding to the Ti–Kα transition, while a secondary peak at 4.93 keV is attributed to the Ti–Kβ transition. The intensity ratio between the Kα and Kβ lines is consistent with the expected theoretical values for titanium, indicating the absence of overlapping contributions from other elements.

Additionally, lower-intensity peaks are observed in the middle region of the spectrum, assigned to the K–Kα and Ca–Kα transitions at 3.31 and 3.69 keV, respectively. The presence of these elements does not indicate external contamination; instead, it reflects the chemical signature of the biological precursor (*Annona muricata* extract). The detection of K and Ca suggests that inorganic mineral residues from the extract remain adsorbed on the nanoparticle surface, potentially acting as stabilizing or capping agents. Quantitative analysis revealed a TiO_2_ content of 85.27%. In addition, small amounts of other compounds were detected, including P_2_O_5_ (2.72%), CaO (0.38%), K_2_O (0.28%), and SiO_2_ (0.28%). These minor components are likely attributed to the co-precipitation of naturally occurring minerals present in the *Annona muricata* extract during the hydrolysis of the TiCl_4_ precursor [43,44].

### 3.2. Open Circuit Potential

**Figure 4:**
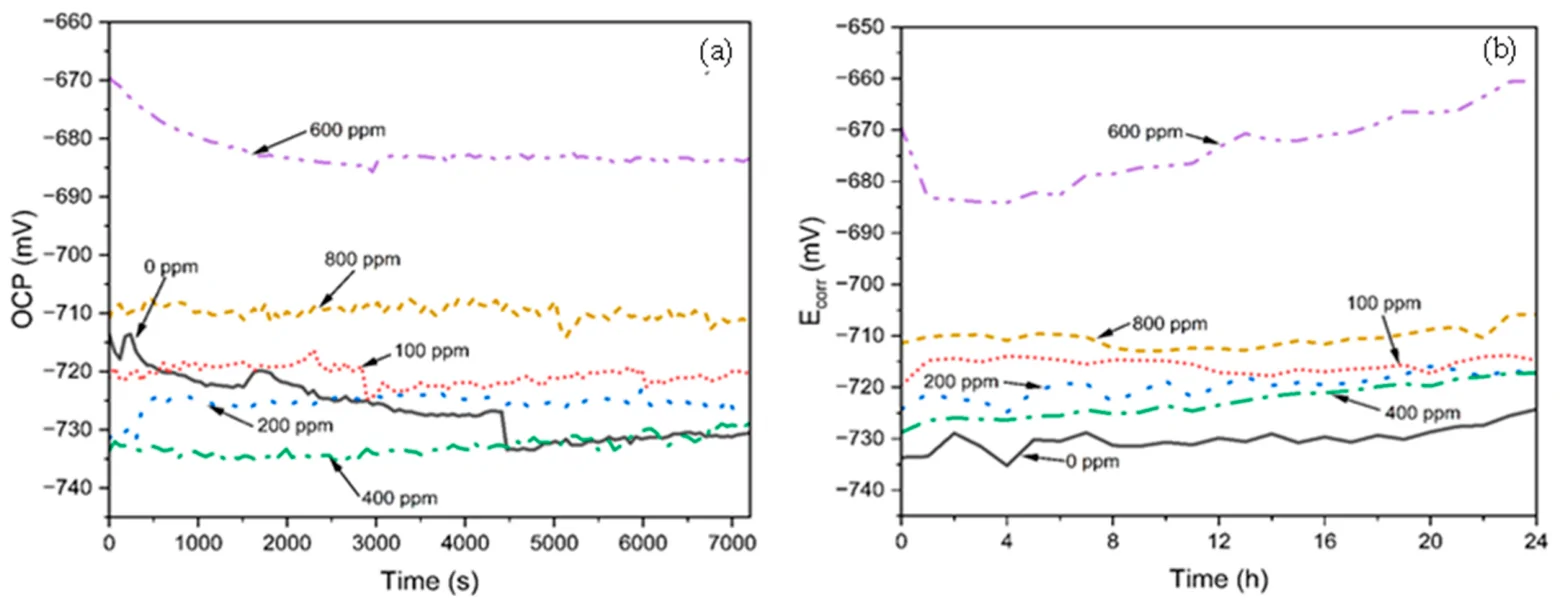
Effect of the TiO_2_ NPs concentration on the *E_corr_* value for 1018 carbon steel in CO_2_-saturated 3.5% NaCl solution during (**a**) first 7200 s, and (**b**) from 2 to 24 h.

The temporal evolution of the open-circuit potential at various TiO_2_ nanoparticle concentrations is depicted in Figure 4a. At all tested concentrations, the OCP shifts towards more positive values relative to the blank solution. This shift into the noble direction suggests the formation of a protective film of TiO_2_ nanoparticles adsorbed on the metal surface, which partially blocks active sites and reduces steel dissolution in the CO_2_-saturated medium. Conversely, the blank solution displays unstable behavior, with a progressive shift towards more negative potentials, from −715 mV to approximately −730 mV during the first two hours. This trend indicates ongoing active dissolution of the steel surface, likely due to the lack of a stable protective layer during the initial stages of immersion.

Upon the addition of 100, 200, and 400 ppm of TiO_2_ NPs, the OCP stability was improved, with reduced fluctuations observed as compared to the blank solution and potential values ranging between −720 and −740 mV. At a concentration of 600 ppm, more noble potentials were rapidly achieved, stabilizing at approximately −685 mV, corresponding to an ennoblement of about 45 mV relative to the blank. This behavior suggests that 600 ppm is the optimal concentration for promoting the formation of a compact and protective barrier layer.

The stability of the protective film and its electrochemical evolution were evaluated over a 24 h immersion period, as shown in Figure 4b. At a concentration of 600 ppm, a continuous and gradual shift towards more noble potentials was observed throughout the entire exposure time. This behavior indicates a dynamic evolution of the protective layer, suggesting that TiO_2_ nanoparticles at this concentration undergo progressive rearrangement, leading to the gradual blocking of pores and active sites on the steel surface. Such a trend is characteristic of increasing corrosion resistance with time due to the formation of a more compact and protective barrier.

In contrast, at 800 ppm, a quasi-steady state was reached after the initial adsorption stage, both during the first two hours and over the 24 h immersion period. Although the system remained stable, the recorded potentials were less noble than those observed at 600 ppm. This behavior may be attributed to the agglomeration of excess TiO_2_ nanoparticles, which reduces the effective surface coverage and introduces defects in the protective film, ultimately decreasing the inhibition efficiency [45,46].

### 3.3. Potentiodynamic Polarization Curves

**Figure 5:**
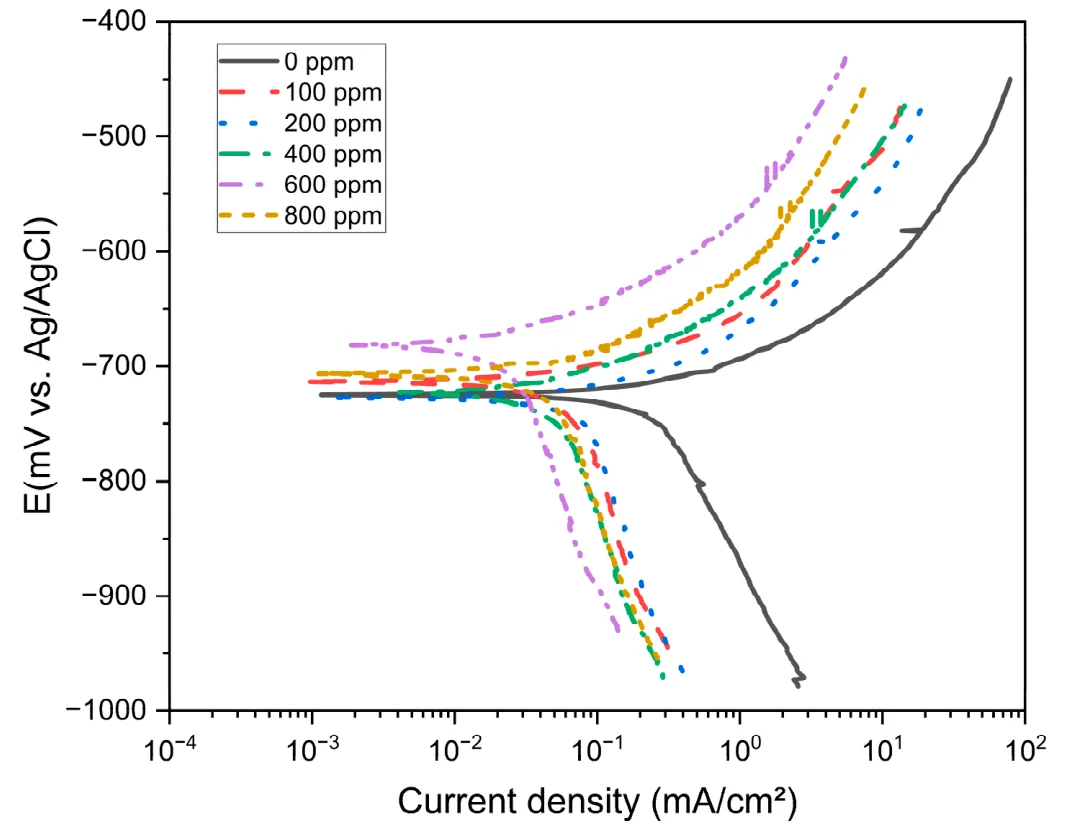
Effect of the TiO_2_ NPs concentration on the polarization curves for 1018 carbon steel in a CO_2_-saturated 3.5% NaCl solution.

The potentiodynamic polarization behavior of the 1018 carbon steel in the uninhibited and inhibited electrolytes is presented in Figure 5. The anodic branches consistently display a lack of passivation, confirming a purely active dissolution mechanism. As expected, the uninhibited sample recorded the highest current density. The introduction of TiO_2_ nanoparticles systematically shifted both the anodic and cathodic branches toward lower current densities, signifying a substantial mitigation of the corrosion rate. This inhibitory effect was maximized at 600 ppm, where a notable suppression of the cathodic kinetics was observed. However, increasing the concentration to 800 ppm caused a shift back to higher current densities, indicating a decrease in inhibition efficiency likely due to nanoparticle agglomeration. Furthermore, the maximum displacement of the corrosion potential relative to the blank remained below the 85 mV threshold. In accordance with established criteria [47], this response classifies the TiO_2_ nanoparticles as a mixed-type inhibitor with a predominant cathodic influence, effectively retarding the corrosion process by simultaneously blocking anodic and cathodic active sites on the metal surface [48].

Table 1 summarizes the electrochemical parameters, including corrosion current density (*i_corr_*), corrosion potential (*E_corr_*), anodic (*β_a_*) and cathodic (*β_c_*) Tafel slopes, as well as surface coverage (θ) and inhibition efficiency (IE). The uninhibited system established a baseline corrosion rate with an *i_corr_* of 0.234 mA/cm^2^, serving as reference. With the addition of the TiO_2_ NPs, a progressive decrease in *i_corr_* values is observed as the TiO_2_ NPs concentration increases. In particular, at 600 ppm, an *i_corr_* value of 0.022 mA/cm^2^ was obtained, corresponding to a reduction in one order of magnitude compared to the blank and resulting in a maximum inhibition efficiency of 91%. However, when the TiO_2_ nanoparticle concentration was increased to 800 ppm, the current density rose, leading to a decrease in inhibition efficiency to 82%. These quantitative results are in excellent agreement with the recent literature. For instance, encapsulated TiO_2_ nanocomposites-such as titania dispersed within polymeric nanogels-have demonstrated potentiodynamic inhibition efficiencies exceeding 91% due to the formation of a highly protective barrier film [49]. This increase in current density supports the previously proposed negative effect of nanoparticle agglomeration, which diminishes the effective surface coverage and introduces defects in the protective film. Furthermore, the anodic and cathodic Tafel slope values confirm that TiO_2_ nanoparticles behave as a mixed-type inhibitor, suppressing both the anodic dissolution of 1018 carbon steel and the primary cathodic reactions inherent to CO_2_ corrosion, namely the direct reduction of carbonic acid and proton reduction [50].

### 3.4. Adsorption Isotherm

As previously indicated, the decrease in the corrosion rate of 1018 carbon steel in a CO_2_-saturated 3.5% NaCl solution at 50 °C is associated with the adsorption of TiO_2_ nanoparticles onto the metal surface.

To elucidate the interaction mechanism between the green-synthesized TiO_2_ nanoparticles and the metallic substrate, the surface coverage data (θ) were evaluated. Conventionally, these experimental data are fitted to the linear Langmuir adsorption isotherm, which assumes a uniform monolayer with no lateral interactions among the adsorbed species. However, as demonstrated by Kokalj [51], this linear mathematical approach is inadequate and yields inaccurate adsorption equilibrium constants ($K_{ads}$) when significant inter-adsorbate interactions are present, often leading to a non-linear behavior at low concentrations.

**Figure 6:**
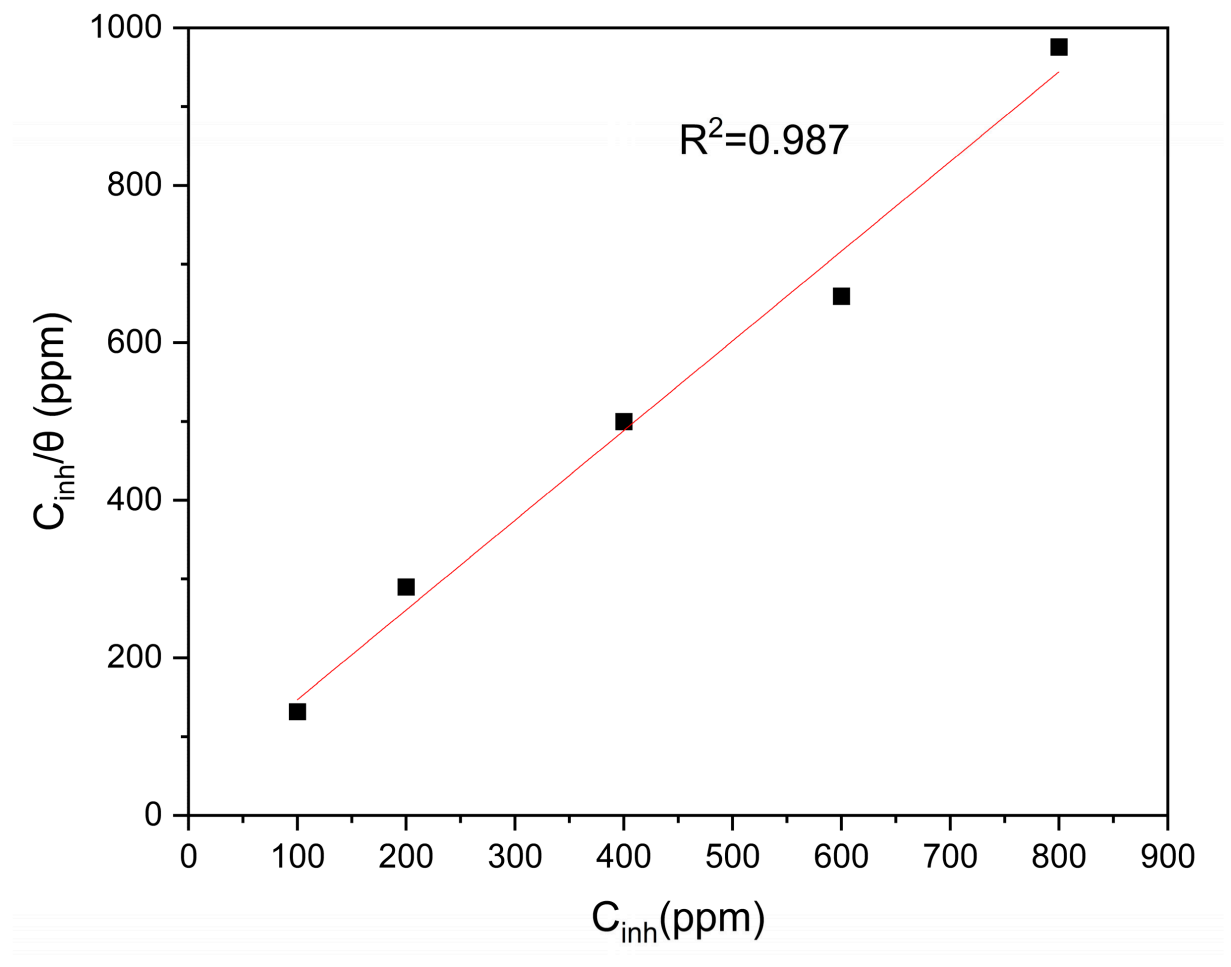
Non-linear fit of the experimental data using Kokalj’s general-purpose Type-I adsorption isotherm for 1018 carbon steel in a CO_2_-saturated 3.5% NaCl solution containing TiO_2_ NPs.

Given that TiO_2_ nanoparticles exhibit strong tendencies to interact laterally and agglomerate via Van der Waals forces at higher concentrations (as supported by our SEM and EIS results at 800 ppm), the conventional linear Langmuir model was discarded. Instead, the experimental data were fitted using the general-purpose Type-I adsorption isotherm proposed by Kokalj:

(3) $$\frac{C_{inh}}{\theta }=\frac{1}{K_{ads}}+{mC}_{inh}+c_{1}\left[exp\left(-\frac{C_{inh}}{c_{2}}\right)-1\right]$$

where m, $c_{1}$, and $c_{2}$ are empirical fitting parameters. A non-linear regression was performed (Figure 6) with the slope restricted to m=1 to accurately capture the curvature of the $C_{inh}/\theta$ plot up to a complete monolayer. This mathematically rigorous fit yielded an outstanding correlation (R^2^ = 0.987) and a corrected equilibrium constant of 0.030 ppm^−1^.

Subsequently, the standard free energy of adsorption ($\Delta G_{ads}^{\circ }$) was calculated utilizing the fundamental thermodynamic equation:

(4) $${∆G}_{ads}^{\circ }=-RTln\left({1\times 10}^{6}K_{ads}\right),$$

In this equation, R represents the universal gas constant, T is the absolute temperature, and the factor 1 × 10^6^ denotes the concentration of water in the solution expressed in mg/L. The non-linear fit yielded a ${∆G}_{ads}^{{}^{\circ}}$ of −27.73 kJ/mol. The negative value of ${∆G}_{ads}^{{}^{\circ}}$ confirms that the adsorption of the nanoparticles is a thermodynamically spontaneous process [52]. Furthermore, the magnitude of $\Delta G_{ads}^{\circ }$ supports a mixed physisorption–chemisorption mechanism [53]. This suggests that adsorption occurs through two sequential stages. Initially, TiO_2_ nanoparticles are physically adsorbed onto the metal surface via electrostatic interactions and Van der Waals forces. This is followed by chemical adsorption, likely promoted by the presence of inorganic mineral residues derived from the *Annona muricata* extract. As evidenced by the XRF analysis, these species remain attached to the nanoparticle surface as capping agents, facilitating coordination through the donation of lone pair and π electrons to the vacant d-orbitals of Fe atoms. Overall, this combined adsorption mechanism leads to the formation of a stable and compact protective film, which effectively limits the diffusion of aggressive species toward the metal surface [54].

### 3.5. Linear Polarization Resistance Experiments

**Figure 7:**
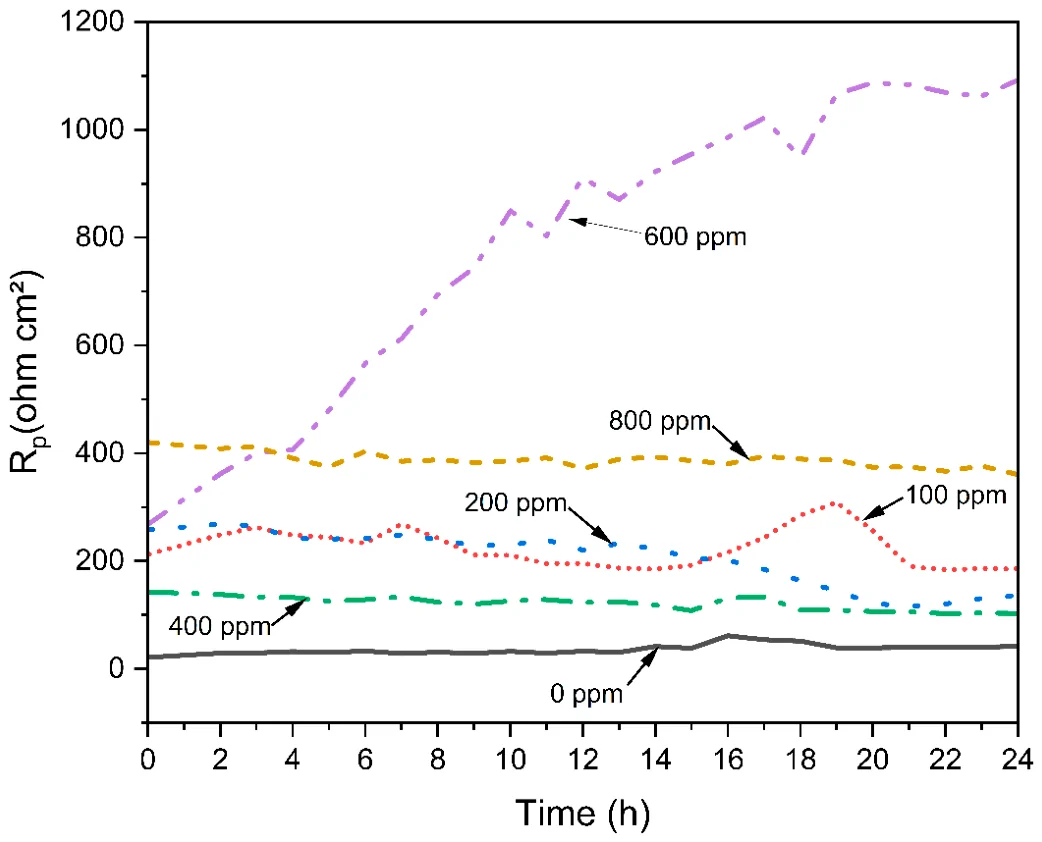
Effect of the TiO_2_ NPs concentration on the R_p_ value for 1018 carbon steel in the CO_2_-saturated 3.5% NaCl solution.

Figure 7 shows the variation in the linear polarization resistance value (R_p_) over a 24 h immersion period for 1018 carbon steel in the CO_2_-saturated 3.5% NaCl solution at different TiO_2_ nanoparticle concentrations. The uninhibited sample exhibited the lowest R_p_ values, remaining nearly constant at around 50 ohm cm^2^, which reflects a high charge transfer rate and, consequently, a higher corrosion rate. The addition of TiO_2_ NPs resulted in an increase in R_p_ value, attributed to their adsorption on the metal surface and the formation of a protective corrosion product layer. At concentrations of 100, 200, and 400 ppm, a moderate rise in R_p_ was observed, with values ranging between 100 and 300 ohm cm^2^, although no significant time-dependent increase was detected. In contrast, the system containing 600 ppm showed a more pronounced response, with R_p_ increasing from approximately 280 ohm cm^2^ to nearly 1100 ohm cm^2^ after 24 h, indicating the gradual growth and densification of the protective film.

Based on the Stern–Geary relationship, polarization resistance is inversely related to the corrosion current density. Thus, the elevated R_p_ values at 600 ppm suggest a considerable reduction in the corrosion current density value. This behavior is associated with the continuous adsorption and compaction of TiO_2_ nanoparticles, which progressively block active sites, seal surface defects, and improve the uniformity of the protective layer, thereby hindering the diffusion of aggressive species towards the metal surface [55].

### 3.6. Electrochemical Impedance Spectroscopy Tests

**Figure 8:**
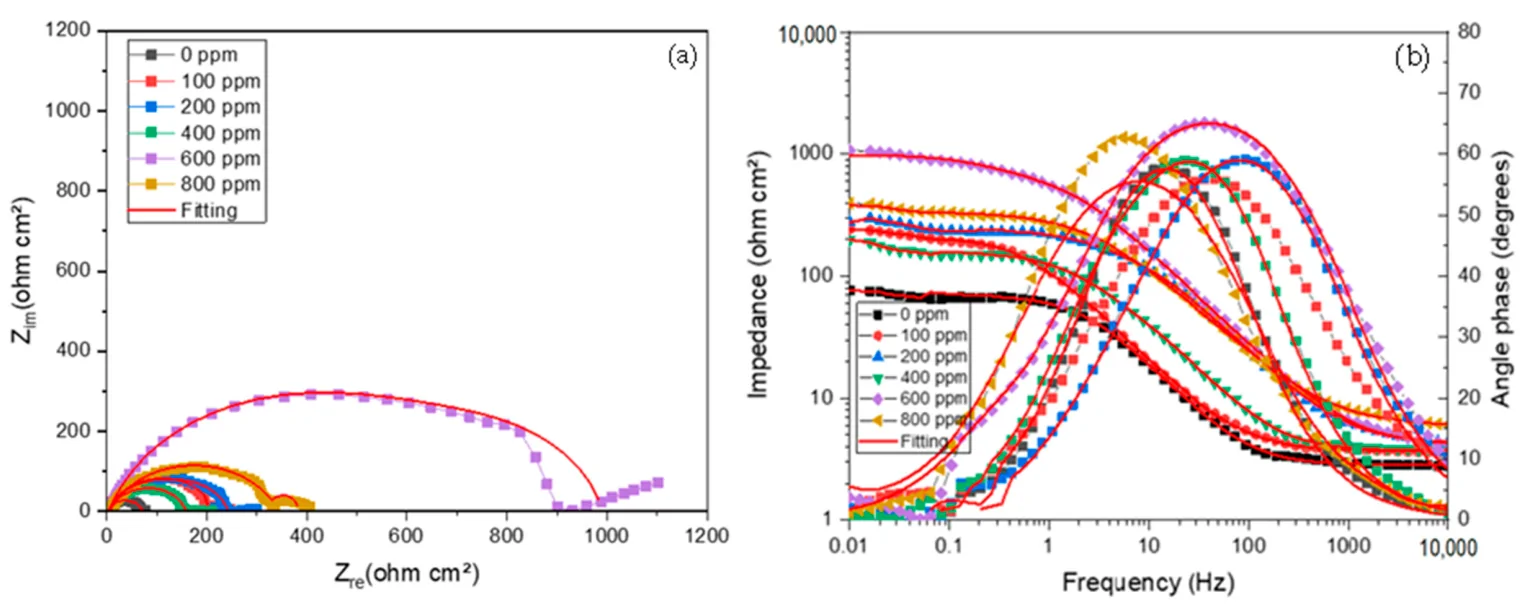
Effect of the TiO_2_ NPs concentration on the (**a**) Nyquist and (**b**) Bode diagrams for 1018 carbon steel in the CO_2_-saturated 3.5% NaCl solution.

To elucidate the corrosion mechanism of 1018 carbon steel in a CO_2_-saturated 3.5% NaCl solution, both in the absence and presence of different concentrations of TiO_2_ nanoparticles as a corrosion inhibitor, electrochemical impedance spectroscopy was employed. The corresponding Nyquist and Bode plots are presented in Figure 8. As shown in Figure 8a, the Nyquist plot for the uninhibited (blank) solution exhibits a single depressed capacitive loop over the entire frequency range. Although it appears as a single semicircle, its depressed and asymmetric shape suggests the presence of overlapping time constants at the metal/solution interface. With the addition of TiO_2_ nanoparticles, the Nyquist plots do not exhibit two clearly resolved semicircles; rather, they present a highly depressed capacitive loop at high-to-medium frequencies followed by a low-frequency tail. The depressed capacitive loop represents the charge transfer process and the electrical double layer capacitance, which deviates from ideal behavior due to the surface roughness and energetic heterogeneity of the corroding steel. The emerging low-frequency tail corresponds to the relaxation process of the biogenic TiO_2_ protective film and the hindered diffusion of species through its porous structure. Notably, the diameter of the primary depressed semicircle (R_ct_) increases progressively with nanoparticle concentration, reaching its maximum at 600 ppm.

This increase in the semicircle diameter, which is proportional to the charge transfer resistance value (R_ct_), indicates the formation of a stable and protective layer on the metal surface. This layer is attributed to the adsorption of TiO_2_ nanoparticles onto active sites, creating a barrier that hinders the diffusion of aggressive species such as Cl^−^ and HCO_3_^−^ towards the metal surface [56]. At a concentration of 800 ppm, a reduction in the diameter of the capacitive semicircle is observed. This behavior suggests possible nanoparticle agglomeration at higher concentrations, driven by strong Van der Waals interactions, which leads to the formation of defects within the protective layer. These defects diminish the effectiveness of the film and facilitate the penetration of corrosive species. This observation is consistent with the overdosing and agglomeration effects previously identified in the OCP and LPR results.

Figure 8b shows the Bode plots, including both the impedance modulus and phase angle. In the low-frequency region, the impedance modulus increases by approximately one order of magnitude at a TiO_2_ nanoparticle concentration of 600 ppm compared to the blank, indicating a significant improvement in the overall corrosion resistance. In contrast, at high frequencies, all curves converge to similar values, confirming that the solution resistance (R_s_) remains essentially unchanged and that the observed differences are primarily due to interfacial phenomena.

The phase angle plots reveal an increase in both the peak width and the maximum phase angle with increasing TiO_2_ nanoparticle concentration, reaching a value close to 70° at 600 ppm. Furthermore, the distinct broadening and asymmetry of the phase angle peak across the medium and high-frequency regions suggest the formation of a protective corrosion product film and the presence of two closely spaced (overlapping) time constants. This behavior perfectly justifies the use of an equivalent circuit with two distinct interfaces: one associated with the adsorbed nanoparticle film and the other corresponding to the electrical double layer at the steel surface.

**Figure 9:**
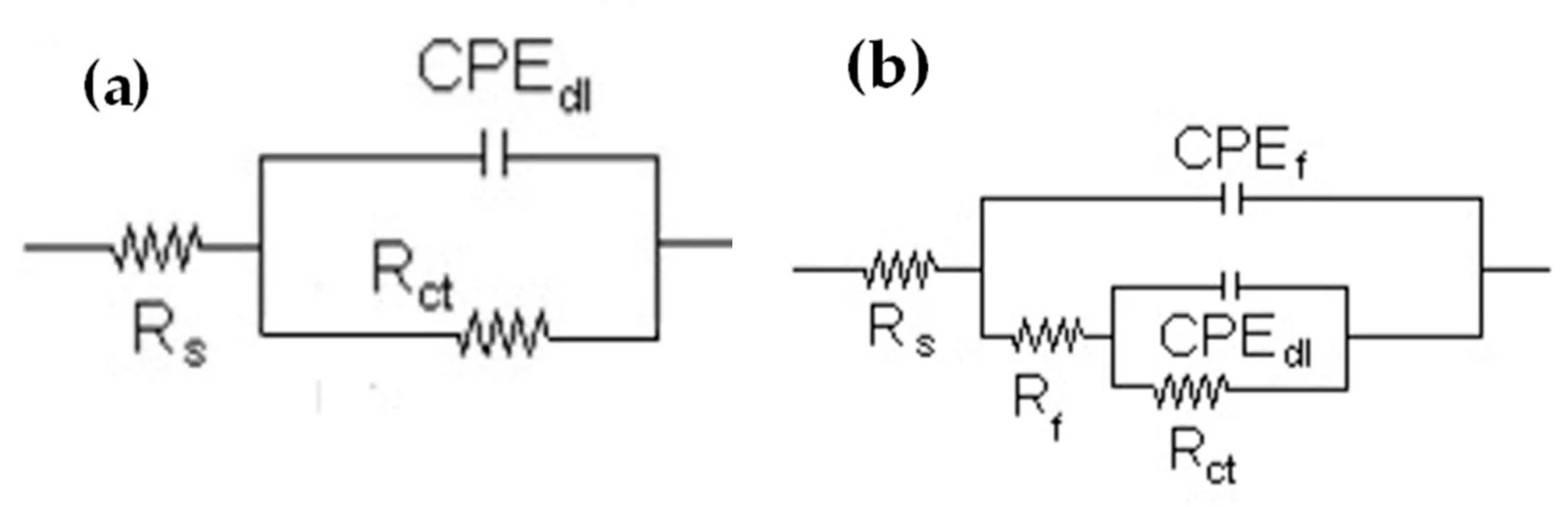
Electrical equivalent circuits used to fit the EIS data for (**a**) uninhibited and (**b**) inhibited NaCl solution.

The quantitative analysis of the impedance spectra was carried out by fitting the experimental data to the electrical equivalent circuits (EECs) illustrated in Figure 9. The proposed two-time-constant circuit (Figure 9b) is a well-established physical model in the literature for describing the charge transfer and relaxation processes of metal interfaces covered by protective inhibitor films [57]. To accurately model the non-ideal capacitive behavior arising from surface roughness and energetic heterogeneities inherent to the corrosion process [58], pure capacitors were substituted with Constant Phase Elements (CPE). The impedance of a CPE is defined as:

(5) $$Z_{CPE}=Y^{-1}{\left(i\omega \right)}^{-n},$$

where Y is the electrical admittance, *i* is the imaginary unit (√−1), ω is the angular frequency (ω = 2πf), *f* is the frequency and *n* has the meaning of a phase shift [59]. The electrochemical parameters obtained from the fitting procedure are summarized in Table 2. The inhibition efficiency (I.E.) was calculated using the following equation:

(6) $$I.E.\left(\%\right)=\frac{R_{ct2}-R_{ct1}}{R_{ct2}}\times 100,$$

where the charge transfer resistance with and without inhibitor are denoted by $R_{ct2}$ and $R_{ct1}$ respectively. A clear increase in the R_ct_ value is observed as the TiO_2_ nanoparticle concentration increases. This occurs because, as the steel dissolution rate decreases, the amount of Fe^2+^ ions crossing the electrochemical double layer also decreases, leading to a reduction in the conductivity of this layer. To accurately evaluate the interfacial behavior, the non-ideal admittance parameter (Y_dl_) of the CPE was mathematically converted into the effective double-layer capacitance (C_dl_) using the Hsu-Mansfeld equation, which is highly suitable for time-constant distributions across protective films [60]:

(7) $$C_{dl}=Y_{dl}^{1/n_{ct}}{(R}_{ct}^{\frac{{1-n}_{ct}}{n_{ct}}}),$$

where Y_dl_ is the magnitude of the double-layer CPE, n_ct_ is its corresponding phase shift parameter, and R_ct_ is the charge transfer resistance. As seen in Table 2, the calculated $C_{dl}$ values decrease significantly with an increase in the TiO_2_ nanoparticle concentration. Physically, the double-layer capacitance can be modeled by the Helmholtz expression [61]:

(8) $$C_{dl}=\left(\frac{{\varepsilon \varepsilon }_{0}}{\lambda }\right)A,$$

where ε_0_ is the vacuum permittivity, ε is the dielectric constant of the medium, λ stands for the double electrochemical layer thickness, and A is the active electrode surface area. A decrease in the dielectric constant of the medium can occur as the TiO_2_ nanoparticles are adsorbed on the metal surface, or an increase in the double-layer thickness. The adsorption of TiO_2_ nanoparticles effectively displaces water molecules from the metallic interface. Because these nanoparticles possess a significantly larger atomic radius compared to water molecules, the thickness of the electrical double layer, λ, in Equation (8) increases, leading to the observed reduction in interfacial capacitance. The increase in the film resistance, R_f_, is observed due to an increase in its thickness as the TiO_2_ nanoparticles are adsorbed. In addition to this, it can be seen that the inhibitor efficiency increases as the TiO_2_ nanoparticle increases, reaching its maximum value at a concentration of 600 ppm in agreement with the results given in Table 1.

### 3.7. Surface Morphology Analysis

**Figure 10:**
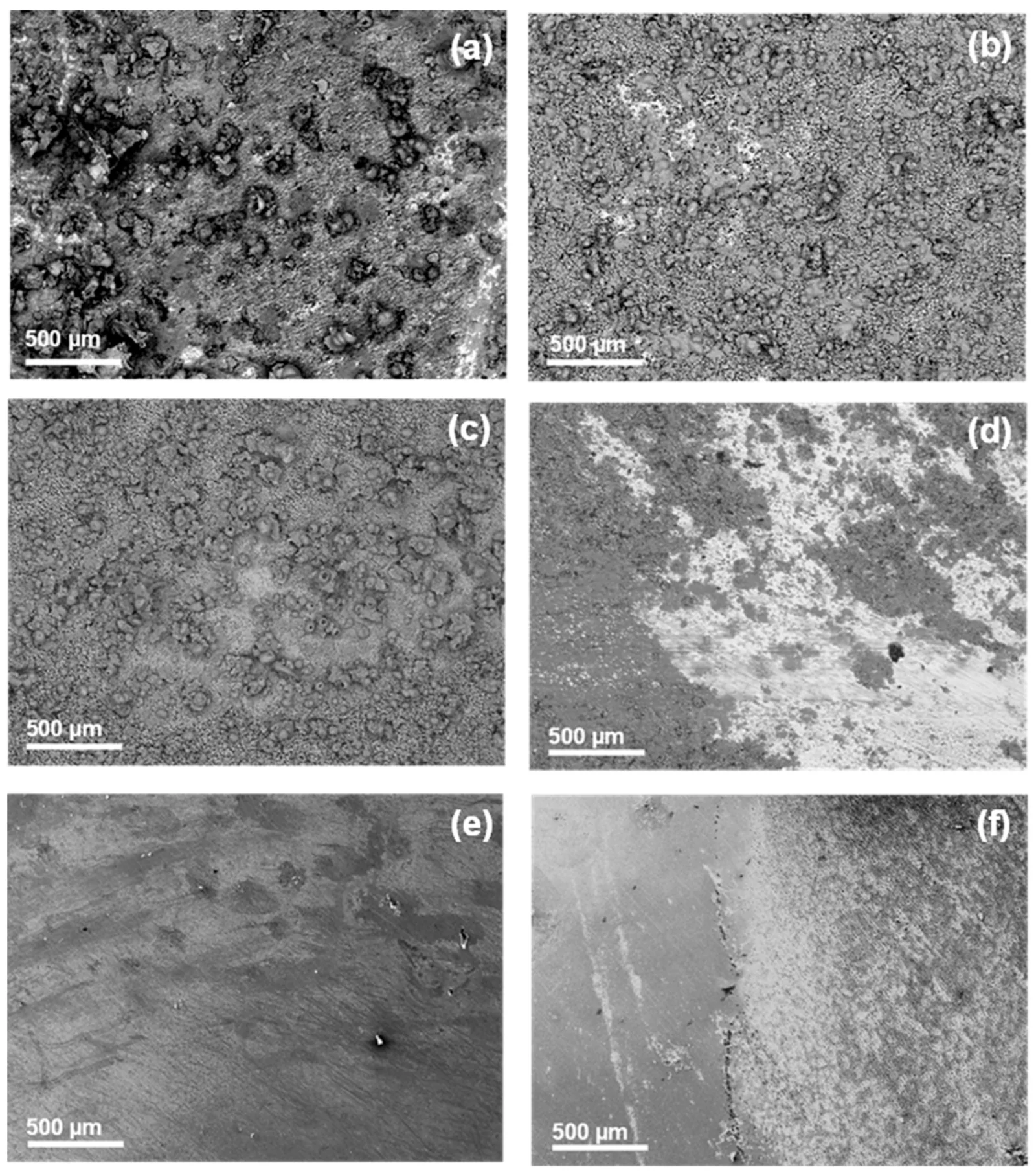
SEM micrographs of 1018 carbon steel corroded in a CO_2_-saturated 3.5% NaCl solution containing (**a**) 0, (**b**) 100, (**c**) 200, (**d**) 400, (**e**) 600 and (**f**) 800 ppm of TiO_2_ NPs.

The surface morphology of 1018 carbon steel after 24 h of immersion in a CO_2_-saturated 3.5% NaCl solution at 50 °C was examined by scanning electron microscopy (SEM). The corresponding micrographs are shown in Figure 10. Figure 10a depicts the sample in the absence of TiO_2_ nanoparticles, where a highly rough and severely degraded surface is observed as a result of corrosion. This deterioration is promoted by the combined action of carbonic acid and chloride ions present in the solution. With the addition of TiO_2_ nanoparticles at concentrations of 100, 200, and 400 ppm (Figure 10b–d), a noticeable reduction in surface damage and roughness is observed, indicating partial mitigation of the corrosive attack. At 600 ppm (Figure 10e), the surface appears significantly smoother, more uniform, and largely free of deep pits. However, when the concentration is further increased to 800 ppm (Figure 10f), localized pitting corrosion becomes evident alongside areas featuring large, irregular precipitates. Following the established adsorption mechanism, this behavior indicates that an excessive TiO_2_ nanoparticle concentration promotes severe agglomeration driven by high surface energy. This agglomeration disrupts the uniform assembly of the nanoparticles, leading to non-uniform surface coverage and the formation of structural defects within the protective film. Ultimately, these defects act as active anodic sites that facilitate the localized ingress of the corrosive medium, thereby reducing the overall inhibition efficiency of the system at higher concentrations.

**Figure 11:**
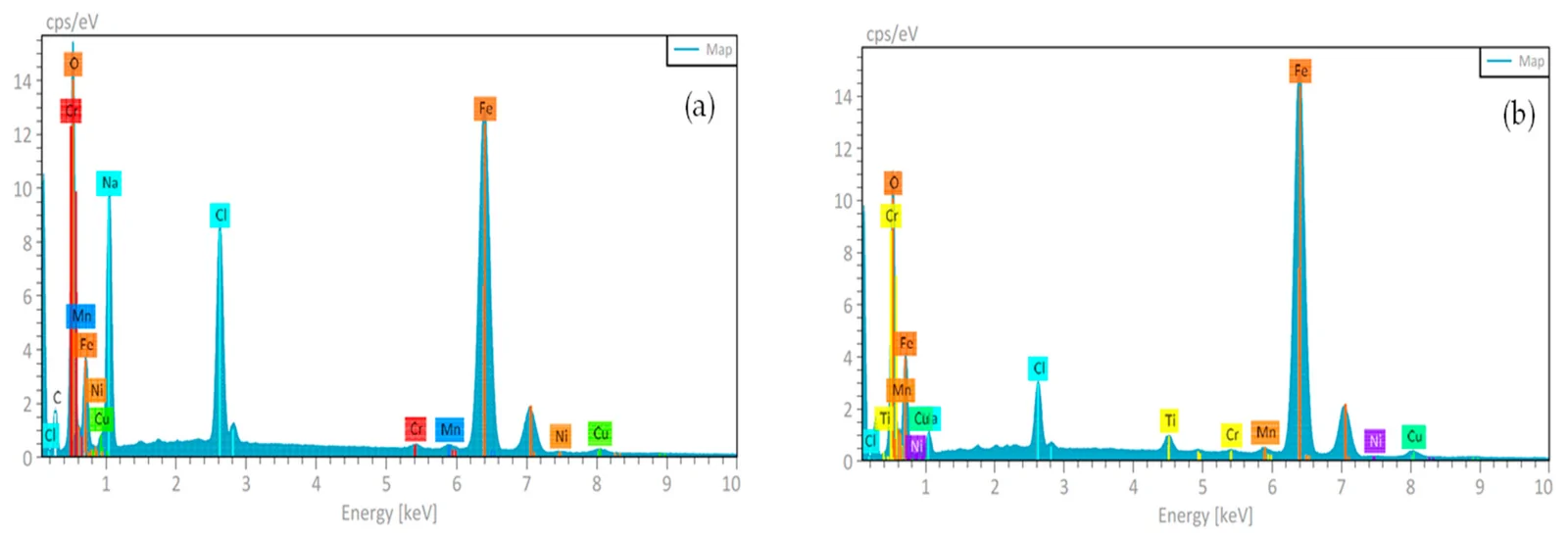
EDS spectra of the 1018 carbon steel surfaces after 24 h of immersion in the CO_2_-saturated 3.5% NaCl solution: (**a**) 0 ppm, and (**b**) 600 ppm of green-synthesized TiO_2_ NPs.

Energy-dispersive X-ray spectroscopy (EDS) analysis was performed on the corroded samples, in the absence and presence of TiO_2_ NPs, to experimentally verify the chemical composition of the corrosion products and corroborate the SEM observations. Figure 11 presents the comparative EDS spectra for the uninhibited sample and the sample with optimal inhibition at 600 ppm. For the uninhibited 1018 carbon steel (Figure 11a), the EDS spectrum and its corresponding quantification (Table 3) reveal intense elemental signals for iron (Fe), carbon (C), and oxygen (O), reaching approximately 39.46 at.% of O and 18.27 at.% of C. The simultaneous presence and high relative intensity of the C and O peaks strongly suggest a stoichiometric ratio consistent with the formation of an FeCO_3_ layer, which constitutes the main and thermodynamically anticipated corrosion product in CO_2_-saturated environments [62]. As observed in the previous SEM analysis, this native carbonate layer is highly porous, failing to provide adequate protection, as evidenced by the significant retention of penetrating aggressive species like Na (17.29 at.%) and Cl (5.02 at.%).

In contrast, the EDS spectrum of the surface inhibited with 600 ppm of TiO_2_ NPs (Figure 11b) displays characteristic titanium (Ti) emission peaks at approximately 0.45 keV and 4.5 keV, with a quantified concentration of 1.06 at.% (1.42 wt.%). Notably, the carbon signal drops below mapping detection limits, and the presence of penetrating Cl ions is drastically reduced to 3.38 at.%. The detection and quantification of Ti on the metallic surface, coupled with the suppression of widespread FeCO_3_ precipitation, provides direct physical evidence of the successful adsorption and incorporation of the TiO_2_ NPs into the interface. This elemental verification is in excellent agreement with the thermodynamic calculations and electrochemical impedance results, confirming that the nanoparticles effectively create a robust barrier against aggressive H_2_CO_3_ and Cl^−^ species.

### 3.8. Corrosion Inhibition Mechanism

**Figure 12:**
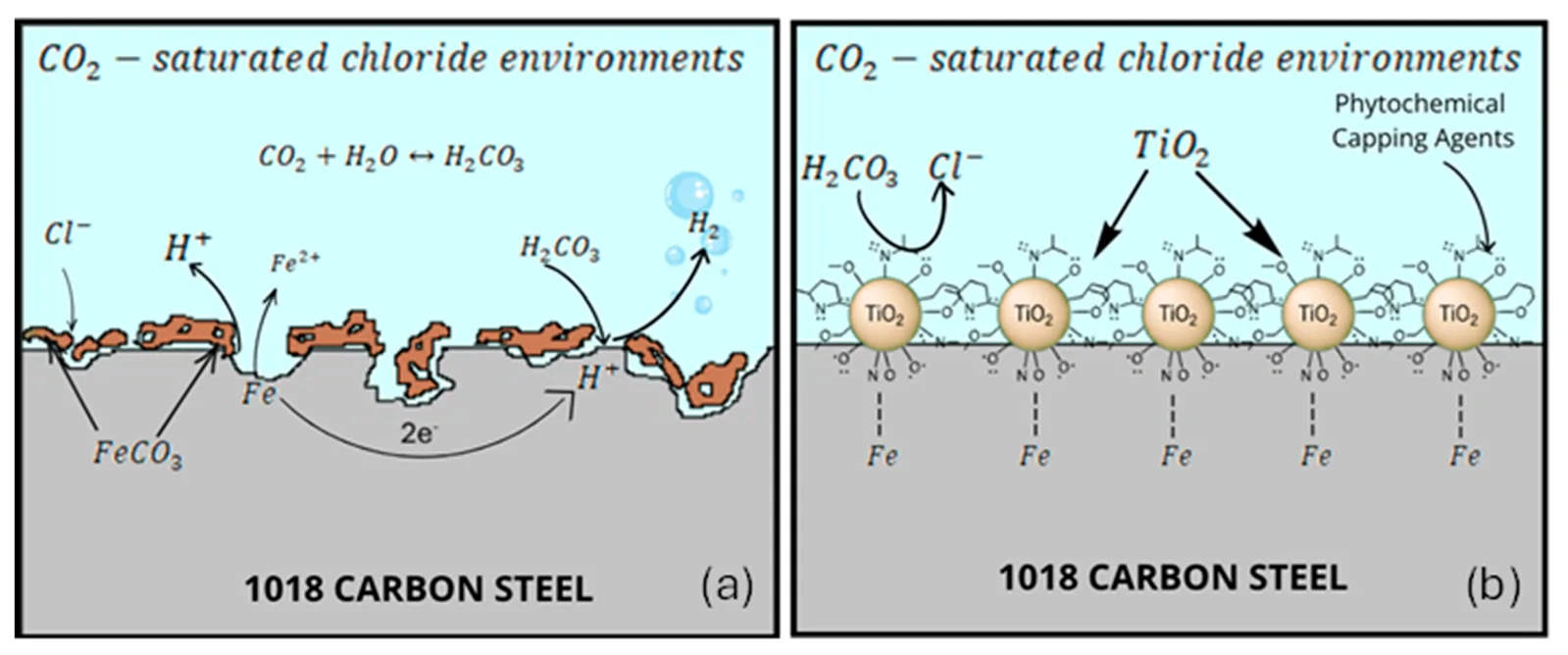
Schematic representation of the corrosion mechanism of 1018 carbon steel in CO_2_-saturated 3.5% NaCl solution: (**a**) Active dissolution and localized attack in the uninhibited medium, and (**b**) formation of a 3D protective barrier via mixed physisorption–chemisorption of green-synthesized TiO_2_ nanoparticles.

Based on the electrochemical results, thermodynamic calculations, and surface characterization, the proposed corrosion inhibition mechanism for 1018 carbon steel in the CO_2_-saturated 3.5% NaCl solution is illustrated in the schematic diagram in Figure 12.

In the absence of the inhibitor, as shown in Figure 12a, the continuous purging of CO_2_ leads to the formation of carbonic acid H_2_CO_3_, which lowers the pH and acts as the primary corrosive species. The active anodic dissolution of iron is accelerated (Equation (9)), while the cathodic sites support the electrochemical reduction in both $H^{+}$ ions and intact H_2_CO_3_ molecules to evolve hydrogen gas (Equations (10) and (11)).

(9) $$Fe\to Fe^{2+}+2e^{-}$$

(10) $${2H}^{+}+{2e}^{-}\to H_{2}$$

(11) $${2H}_{2}{CO}_{3}+{2e}^{-}\to \;H_{2}+{2HCO}_{3}^{-}$$

The aggressive chloride ions (Cl^−^) present in the bulk solution easily penetrate any poorly adhered FeCO_3_ corrosion products, leading to the severe and highly rough localized degradation observed in the SEM micrographs [63].

Conversely, upon the addition of the optimal concentration of green-synthesized TiO_2_ nanoparticles, as illustrated in Figure 12b, the degradation is effectively suppressed through a mixed-type inhibition mechanism. The adsorption of these nanoparticles onto the metallic substrate follows a combined physisorption and chemisorption process, as evidenced by the standard free energy of adsorption ($\Delta G_{ads}^{\circ }=-27.73\;\mathrm{kJ}/\mathrm{mol}$). Initially, the nanoparticles physiosorbed onto the charged metal surface via electrostatic interactions. Subsequently, the phytochemicals derived from the *Annona muricata* extract, which act as stabilizing capping agents on the TiO_2_ surface, facilitate chemisorption. These organic molecules possess heteroatoms with lone pairs of electrons that can coordinate with the vacant d-orbitals of the surface iron atoms [64].

This synergistic interaction creates a dense, highly stable protective film. The organic capping agents ensure robust adhesion to the steel, while the inorganic TiO_2_ core provides an impermeable physical barrier. This barrier significantly increases the charge transfer resistance and effectively restricts the diffusion of H_2_CO_3_ and Cl^−^ toward the metallic interface, thereby reducing the corrosion current density by nearly an order of magnitude [65].

## 4. Conclusions

This research successfully demonstrated the biogenic synthesis of TiO_2_ nanoparticles mediated by *Annona muricata* aqueous extract, validating their outstanding performance as eco-friendly corrosion inhibitors for carbon steel infrastructure exposed to sweet corrosion environments (3.5 wt.% NaCl saturated with CO_2_ at 50 °C). The incorporation of nanoparticles into the solution shifted the open-circuit potential toward more noble values, attributed to their progressive adsorption and the formation of a protective surface film. The corrosion current density decreased by one order of magnitude in the presence of TiO_2_ nanoparticles. The inhibition efficiency increased with concentration, reaching a maximum of 91% at 600 ppm, and then decreased to 82% at 800 ppm due to the adverse effects of nanoparticle agglomeration. The nanoparticles adhered to the metal surface through a spontaneous mixed physisorption–chemisorption mechanism, in which phytochemical compounds acted as capping agents, promoting coordination with the Fe d-orbitals. TiO_2_ nanoparticles functioned as a mixed-type inhibitor, influencing both anodic and cathodic electrochemical reactions. The corrosion process remained controlled by charge transfer and was not altered by the presence of the TiO_2_ nanoparticles, although a significant increase in polarization resistance was observed over time. While the uninhibited steel exhibited pronounced susceptibility to active anodic dissolution, the addition of TiO_2_ nanoparticles—particularly at the optimal concentration of 600 ppm—substantially mitigated surface degradation, yielding a smoother and less porous protective film.

## Figures and Tables

**Table 1:** Electrochemical parameters obtained from polarization curves.

TiO_2_ (ppm)	*i_corr_* (mA/cm^2^)	*E_corr_* (mV)	*β_a_* (mV/dec)	*β_c_* (mV/dec)	I.E. (%)	θ
0	0.234 ± 0.01	−722 ± 5	48 ± 1	237 ± 7		
100	0.055 ± 0.002	−714 ± 6	44 ± 2	227 ± 9	76 ± 3	0.76
200	0.073 ± 0.003	−727 ± 6	40 ± 2	235 ± 9	69 ± 3	0.69
400	0.047 ± 0.001	−724 ± 7	67 ± 2	245 ± 6	80 ± 2	0.80
600	0.022 ± 0.001	−679 ± 5	53 ± 2	250 ± 9	91 ± 3	0.91
800	0.042 ± 0.002	−704 ± 6	60 ± 2	251 ± 7	82 ± 2	0.82

**Table 2:** Electrochemical parameters used to fit the EIS results for 1018 carbon steel in a CO_2_-saturated 3.5% NaCl without and with the addition of TiO_2_ NPs.

C_inh_ (ppm)	Chi-Sqr	R_s_ (ohm cm^2^)	R_ct_ (ohm cm^2^)	C_dl_ (µF cm^−2^)	n_ct_	R_f_ (ohm cm^2^)	C_f_ (µF cm^−2^)	n_f_	I.E. (%)
0	3.02 × 10^−3^	2.8 ± 0.08	67 ± 4	1076.3 ± 58	0.9	-	-	-	-
100	6.52 × 10^−4^	3.6 ± 0.09	89 ± 7	638 ± 51	0.9	2.2 ± 0.2	2959.5 ± 222	0.8	23 ± 1
200	4.22 × 10^−4^	4.2 ± 0.11	157 ± 12	271.3 ± 20	0.8	25 ± 2	5584.7 ± 402	0.9	63 ± 2
400	6.82 × 10^−4^	3.7 ± 0.1	216 ± 17	355.9 ± 28	0.9	37 ± 2	3443.9 ± 155	0.8	70 ± 3
600	3.41 × 10^−4^	4.0 ± 0.12	780 ± 47	125.7 ± 8	0.8	207 ± 14	1415.1 ± 93	0.9	91 ± 2
800	3.48 × 10^−4^	5.9 ± 0.16	337 ± 21	190.6 ± 12	0.8	65 ± 4	3063.5 ± 202	0.9	80 ± 3

**Table 3:** Quantitative EDS elemental composition (wt.% and at.%) of 1018 carbon steel surfaces after 24 h of immersion in the CO_2_-saturated 3.5% NaCl solution.

Element	0 ppm TiO_2_ (wt.%)	0 ppm TiO_2_ (at.%)	600 ppm TiO_2_ (wt.%)	600 ppm TiO_2_ (at.%)
Iron (Fe)	41.82	19.05	68.57	43.84
Oxygen (O)	24.82	39.46	20.27	45.23
Carbon (C)	8.63	18.27	-	-
Sodium (Na)	15.63	17.29	2.81	4.37
Chlorine (Cl)	7.00	5.02	3.35	3.38
Titanium (Ti)	-	-	1.42	1.06

## Data Availability

The original contributions presented in this study are included in the article. Further inquiries can be directed to the corresponding author.
